# Protein *N*-Myristoylation Plays a Critical Role in the Endoplasmic Reticulum Morphological Change Induced by Overexpression of Protein Lunapark, an Integral Membrane Protein of the Endoplasmic Reticulum

**DOI:** 10.1371/journal.pone.0078235

**Published:** 2013-11-04

**Authors:** Koko Moriya, Kei Nagatoshi, Yoshimi Noriyasu, Tsuyoshi Okamura, Emi Takamitsu, Takashi Suzuki, Toshihiko Utsumi

**Affiliations:** 1 Applied Molecular Bioscience, Graduate School of Medicine, Yamaguchi University, Yamaguchi, Japan; 2 Department of Biological Chemistry, Faculty of Agriculture, Yamaguchi University, Yamaguchi, Japan; 3 Clinical & Biotechnology Business Unit, Shimadzu Corporation, Kyoto, Japan; Ecole Polytechnique Federale de Lausanne, Switzerland

## Abstract

*N*-myristoylation of eukaryotic cellular proteins has been recognized as a modification that occurs mainly on cytoplasmic proteins. In this study, we examined the membrane localization, membrane integration, and intracellular localization of four recently identified human *N*-myristoylated proteins with predicted transmembrane domains. As a result, it was found that protein Lunapark, the human ortholog of yeast protein Lnp1p that has recently been found to be involved in network formation of the endoplasmic reticulum (ER), is an *N*-myristoylated polytopic integral membrane protein. Analysis of tumor necrosis factor-fusion proteins with each of the two putative transmembrane domains and their flanking regions of protein Lunapark revealed that transmembrane domain 1 and 2 functioned as type II signal anchor sequence and stop transfer sequence, respectively, and together generated a double-spanning integral membrane protein with an N-/C-terminal cytoplasmic orientation. Immunofluorescence staining of HEK293T cells transfected with a cDNA encoding protein Lunapark tagged with FLAG-tag at its C-terminus revealed that overexpressed protein Lunapark localized mainly to the peripheral ER and induced the formation of large polygonal tubular structures. Morphological changes in the ER induced by overexpressed protein Lunapark were significantly inhibited by the inhibition of protein *N*-myristoylation by means of replacing Gly2 with Ala. These results indicated that protein *N*-myristoylation plays a critical role in the ER morphological change induced by overexpression of protein Lunapark.

## Introduction

Protein *N*-myristoylation is the attachment of myristic acid, a 14-carbon saturated fatty acid, to the N-terminal Gly of proteins [1]–[5]. This modification is a well-recognized form of lipid modification that occurs on eukaryotic and viral proteins. In general, myristic acid is cotranslationally attached to the N-terminal Gly residue after removal of the initiating Met. In addition to the cotranslational protein *N*-myristoylation, it is now established that posttranslational *N*-myristoylation can also occur on many caspase-cleavage products in apoptotic cells [6]–[8]. Both cotranslational and posttranslational *N*-myristoylation are catalyzed by *N*-myristoyltransferase (NMT), a member of the GCN5-related *N*-acetyltransferase superfamily of proteins [9]. Many *N*-myristoylated proteins play key roles in regulating cellular structure and function. They include proteins involved in a wide variety of cellular signal transduction pathways, such as protein kinases, phosphatases, guanine nucleotide binding proteins, Ca^2+^ binding proteins, and cytoskeletal regulatory proteins. In addition to the proteins involved in cellular signal transduction pathways, recent studies have revealed that protein *N*-myristoylation occurs on many disease-related proteins [10]–[14]. In many cases, the functions of these *N*-myristoylated proteins are regulated by reversible membrane binding mediated by protein *N*-myristoylation. Thus, protein *N*-myristoylation has been recognized as a protein modification that occurs mainly on cytoplasmic proteins, and only very few integral membrane proteins have been demonstrated to be *N*-myristoylated so far.

In viral proteins, however, protein *N*-myristoylation has been found to occur on integral membrane proteins. It has been demonstrated that the large L envelope protein of the hepatitis B virus (HBV), a polytopic membrane protein, is *N*-myristoylated and this modification is involved in the intracellular retention of this protein and is essential for viral infectivity [15]–[17]. This protein has the exceptional capacity to form two transmembrane topologies via an uncharacterized process of partial post-translational translocation of its pre-S domain across membranes [18]–[20]. HBV L protein with an *N*-myristoylated N-terminal pre-S domain plays critical roles in the viral life cycle by mediating receptor binding during host cell attachment. Thus, protein *N*-myristoylation of HBV L protein plays vital roles in the expression of the specific functions of this protein.

In eukaryotic cellular proteins, only very few integral membrane proteins have been demonstrated to be *N*-myristoylated. One example of an *N*-myristoylated integral membrane protein in eukaryotes is mammalian NADH-cytochrome b(5) reductase (b5R), which is a single-spanning membrane protein with N-exo/C-cyto orientation and is dually targeted to the ER and mitochondrial outer membranes [21], [22]. In b5R, protein *N*-myristoylation is required for the targeting to the mitochondria, because a non-myristoylated mutant is exclusively localized to the ER [23]. It was further revealed that protein *N*-myristoylation interferes with interaction of the nascent chain with the signal recognition particle, so that a portion of the nascent chains escape from cotranslational integration into the ER and can be post-translationally targeted to the mitochondrial outer membrane [24]. Another mammalian integral membrane protein that has been shown to be *N*-myristoylated is dihydroceramide Δ4-desaturase 1 (DES1) [25]. As is the case with b5R, DES1 is dually targeted to the ER and mitochondria, and it was shown that protein *N*-myristoylation favors to localization to the mitochondria [26]. In addition, it was also demonstrated that protein *N*-myristoylation positively affected the enzymatic activity of DES1 [25], [27].

In the present study, we searched for novel human *N*-myristoylated transmembrane proteins using four recently identified human *N*-myristoylated proteins with predicted transmembrane domains. As a result, it was found that protein Lunapark, the human ortholog of yeast protein Lnp1p that has recently been found to be involved in the network formation of endoplasmic reticulum (ER) [28], is an *N*-myristoylated polytopic transmembrane protein. Protein Lunapark belongs to the Lunapark family of proteins. It has been reported that this protein might be involved in the development of limbs and the central nervous system [29]. However, a recent report revealed that Lnp1p, the yeast ortholog of protein Lunapark, is a membrane protein of the ER and is involved in network formation of the ER [28]. Despite the physiological importance of the protein, biochemical characterization of the membrane integration and membrane topology of protein Lunapark has not been performed. In this study, we first characterized the role of two putative transmembrane domains of protein Lunapark in the membrane translocation and memberane topology formation of this protein. Then, we studied the role of protein N-myristoylation occurs on protein Lunapark on the membrane translocation, membrane topology formation, intracellular localization, and physiological function of this protein. As a result, it was revealed that transmembrane domain 1 and 2 functioned as type II signal anchor sequence and stop transfer sequence, respectively, and together generated a double-spanning integral membrane protein with an N-/C-terminal cytoplasmic orientation. It was also found that protein N-myristoylation did not affect membrane translocation, membrane topology formation, and intracellular localization of protein Lunapark. Interestingly however, immunofluorescence staining of HEK293T cells transfected with cDNA encoding protein Lunapark tagged with FLAG-tag at its C-terminus revealed that overexpressed protein Lunapark localized to the peripheral ER and induced the formation of large polygonal tubular structures, and this phenomenon was significantly inhibited by the inhibition of protein *N*-myristoylation by the replacement of Gly2 with Ala. These results indicated that protein *N*-myristoylation plays critical roles in ER morphological changes induced by overexpression of protein Lunapark. To our knowledge, this is the first report clarifying the role of protein *N*-myristoylation in membrane translocation, membrane topology formation, intracellular targeting, and the function of a human *N*-myristoylated polytopic membrane protein.

## Materials and Methods

### Materials

Restriction endonucleases, DNA-modifying enzymes, RNase inhibitor, and Taq DNA polymerase were purchased from Takara Shuzo (Kyoto, Japan). Transdirect insect cell, an insect cell-free protein synthesis system, was obtained from Shimadzu (Kyoto, Japan). RNase was from Boehringer-Mannheim (Germany). [^3^H]Leucine and [^3^H]myristic acid were from GE Healthcare (Piscataway, NJ, USA). ENLIGHTNING was from PerkinElmer (Shelton, CT, USA). The dye terminator cycle sequencing kit was from Applied Biosystems. Anti-FLAG monoclonal antibody, fluorescein isothiocyanate-conjugated anti-mouse IgG antibody, myristoyl-CoA, CHCA, DHB, anti-FLAG M2-Agarose, and FLAG peptide were purchased from Sigma (St. Louis, MO, USA). ER-Tracker Red, Alexa Fluor 594 anti-mouse IgG antibody, and Hoechst 33342 were obtained from Molecular Probes (Eugene, OR, USA). Human cDNAs (FlexiORF Clones), AmpliScribe T7 High Yield Transcription Kit, T7 RiboMAX Express Large Scale RNA Production System were purchased from Promega (Madison, WI, USA). ProteoExtract subcellular proteome extraction kit was from Merck KGaA (Darmstadt, Germany). Other reagents were purchased from Wako Pure Chemical (Osaka, Japan), Daiichi Pure Chemicals (Tokyo, Japan), or Seikagaku Kogyo (Tokyo, Japan) and were of analytical or DNA grade.

### Plasmid construction

Nucleotide sequences of oligonucleotide primers used for the plasmid construction are summarized in Table S1. Plasmid pcDNA3-FL used as the mammalian expression vector was constructed as described previously [30]. For the construction of pcDNA3 plasmids containing C-terminally FLAG-tagged full-length KOP cDNA clones (ARF1-FLAG, SERINC1-FLAG, KIAA1609-FLAG, Lunapark-FLAG, ZZEF1-FLAG), pcDNA3-FL was treated with SgfI and EcoRV and KOP cDNA clones digested with SgfI and PmeI were subcloned into the vector. Plasmid pTD1-FL used for the insect cell-free protein synthesis system was constructed as described previously [30]. For the construction of pTD1 plasmids containing C-terminally FLAG-tagged full-length KOP cDNA clones, pTD1-FL was digested with SgfI and EcoICRI and KOP cDNA clones digested with SgfI and PmeI were subcloned into the vector. Plasmid pBpro-GLC-TNF was constructed as described previously [31]. In this mutant, an N-glycosylation site (Asn-X-Ser) was introduced at amino acid positions 45–47 in the mature domain of tumor necrosis factor (TNF). Construction of plasmids pcDNA3ΔMet-GLC-TNF, pcDNA3pro-GLC-TNF, pcDNA3Lunapark-TM1-GLC-TNF, pcDNA3Lunapark-TM1-G2A-GLC-TNF, pcDNA3Lunapark-TM1/2-GLC-TNF, pcDNA3Lunapark-GLC-TNF, was summarized in Table S2. Plasmid pcDNA3Lunapark-G2A-FLAG was constructed using a Prime STAR Mutagenesis Kit (TAKARA) with two oligonucleotides (Primer-N7 and Primer-C7) as primers and pcDNA3Lunapark-FLAG as a template. Plasmid pcDNA3Lunapark-CtoA-FLAG in which Cys276, Cys279, Cys298, and Cys301 in Lunapark-FLAG were substituted with Ala was constructed using a Prime STAR Mutagenesis Kit (TAKARA) as follows. pcDNA3Lunapark-C276,279A-FLAG was first constructed by PCR using two oligonucleotides (Primer-N8 and Primer-C8) as primers and pcDNA3Lunapark-FLAG as a template. Next, pcDNA3Lunapark-CtoA-FLAG was constructed by PCR using two oligonucleotides (Primer-N9 and Primer-C9) as primers and pcDNA3Lunapark-C276,279A-FLAG as a template. Plasmid pBH14-TNF (previously designated as pBΔ-75-47,-32-1 pro-TNF) was constructed as described previously [32]. Construction of plasmids, pcDNA3EGFP, pcDNA3EGFP-Sec61β, pcDNA3H14-TNF-Δtrm, pcDNA3H14-TNF, pcDNA3H14-TNF-Lunapark-TM2, pcDNA3H14-TNF-Lunapark-ΔTM2, was summarized in Table S2. The DNA sequences of these recombinant cDNAs were confirmed by the dideoxy-nucleotide chain termination method.

### Detection of protein *N*-myristoylation using an insect cell-free protein synthesis system

The cDNAs were subcloned into vector pTD1 (Shimadzu Co.) at a site under the control of the T7 promoter. The mRNAs encoding the cDNAs were prepared using an AmpliScribe T7 High Yield Transcription Kit in accordance with manufacturer's instructions. The translation reaction was carried out using an insect cell-free protein synthesis system (Shimadzu Co.) in the presence of [^3^H]leucine or [^3^H]myristic acid, under conditions recommended by the manufacturer. The mixture (composed of 12.5 µL of insect cell lysate, 7.5 µL of reaction buffer, 0.5 µL of 1 mM leucine-free amino acid mixture, 2.0 µL of [^3^H]leucine (2 µCi) or [^3^H]myristic acid (40 µCi), and 2.5 µL of mRNA (5 µg)) was incubated at 25°C for 6 h. The samples were then analyzed by SDS–PAGE and fluorography.

### *In vitro* synthesis and affinity-purification of protein Lunapark

The mRNA encoding protein Lunapark was synthesized at a 40 µL scale using a T7 RiboMAX Express Large Scale RNA Production System (Promega) in accordance with manufacturer's instructions. After completion of the reaction, 60 µL of 25 mM EDTA was added to the *in vitro* transcription mixture. The mixture was then used as the template for *in vitro* translation. *In vitro* translation was performed at a 1 mL scale using a Transdirect insect cell in the presence of 50 µM myristoyl-CoA in accordance with the manufacturer's instructions. Affinity purification of FLAG-tagged protein was performed as described previously [33].

### MS and MS/MS analysis of *in vitro*-synthesized protein Lunapark

The sample preparation for MS analysis was performed as described previously [33]. MS spectra and MS/MS spectra were acquired in reflectron positive ion mode with an AXIMA-CFR-plus MALDI-TOF MS instrument and an AXIMA-QIT MALDI-QIT-TOF hybrid mass spectrometer (Shimadzu/Kratos, Manchester, UK), respectively.

### Transfection of cells and determination of *N*-myristoylated and *N*-glycosylated proteins

COS-1 (the simian virus 40-transformed African green monkey kidney cell line, American Type Culture Collection) or HEK293T (a human embryonic kidney cell line) cells were maintained in Dulbecco's modified Eagle's medium (DMEM; Gibco BRL) supplemented with 10% fetal calf serum (FCS; Gibco BRL). Cells (2×10^5^) were plated onto 35-mm diameter dishes 1 day before transfection. pcDNA3 constructs (2 µg) containing cDNAs encoding FLAG-tagged, EGFP-tagged, or TNF-fusion proteins were used to transfect each plate of cells along with 4 µL of Lipofectamine (2 mg/mL, Gibco BRL; Barcelona, Spain) in 1 mL of serum-free medium. After incubation for 5 h at 37°C, the cells were refed with serum-containing medium and incubated again at 37°C for 24 h. For determination of protein *N*-myristoylation, the cells were then washed twice with 1 mL of serum-free DMEM and incubated for 6 h at 37°C in 1 mL of DMEM (+2% FCS) containing [^3^H]myristic acid (100 µCi/mL). Subsequently, the cells were washed three times with Dulbecco's phosphate-buffered saline (DPBS) and collected with a cell scraper and then lyzed with 200 µL of RIPA buffer (50 mM Tris-HCl [pH 7.5], 150 mM NaCl, 1% Nonidet P-40, 0.5% sodium deoxycholate, 0.1% sodium dodecyl sulfate (SDS), and proteinase inhibitors) on ice for 20 min. The samples were then analyzed by sodium dodecyl sulfate polyacrylamide gel electrophoresis (SDS-PAGE) and fluorography. To examine the *N*-glycosylation of TNF-mutants, 24 h after transfection, total cell lysates were obtained as described above and 20 µL of the total cell lysate of each group of transfected cells were treated with 10 mU/mL of glycopeptidase F at 37°C for 1 h and then analyzed by Western blotting [31].

### Subcellular fractionation

Subcellular fractionation of COS-1 cells expressing either KIAA1609-FLAG or Lunapark-FLAG was performed by using a ProteoExtract subcellular proteome extraction kit (Merck) as described previously [34]. Briefly, COS-1 cells (2×10^5^) were transfected with 2 µg of pcDNA3KIAA1609-FLAG or pcDNA3Lunapark-FLAG as described earlier and incubated at 37°C for 24 h. After washing twice with ice-cold Wash Buffer, cells were incubated with 0.5 mL of ice-cold Extraction Buffer I at 4°C for 10 min, and then the supernatant was collected and used as a cytosolic fraction. Subsequently, cells were incubated with 0.5 mL of ice-cold Extraction Buffer II at 4°C for 30 min, and then the supernatant was collected and used as a membrane/organelle fraction. The cells were then incubated with 0.5 mL ice-cold Extraction Buffer III at 4°C for 10 min, then the supernatant was collected and used as a nucleic fraction.

### Western blotting

The total cell lysates of each group of transfected cells were prepared 24 h after transfection and resolved by 12.5% SDS-PAGE and then transferred to an Immobilon-P transfer membrane (Millipore; Billerica, MA, USA). After blocking with nonfat milk, the membrane was probed with a specific anti-FLAG or anti-TNF antibody as described previously [35]. Immunoreactive proteins were specifically detected by incubation with horseradish peroxidase-conjugated protein G (Bio-Rad; Hercules, CA, USA). The membrane was developed using enhanced chemiluminescence Western blotting reagent (Amersham Biosciences; Buckinghamshire, UK) and exposed to X-ray film (Eastman Kodak Co.; Rochester, NY, USA).

### SDS-PAGE and fluorography

Samples were denatured by boiling for 3 min in SDS-sample buffer followed by analysis by SDS-PAGE on a 12.5% gel. Thereafter, the gel was fixed and soaked in ENLIGHTNING (PerkinElmer; Waltham, MA, USA) for 20 min. The gel was dried under vacuum and exposed to X-ray film (Kodak) for an appropriate period.

### Immunofluorescence analysis, fluorescence microscopy, and determination of morphological changes of ER

Immunofluorescence analysis and fluorescence microscopic analysis of transfected cells was performed 24 h after transfection [34]. For immunocytochemistry, the cells were washed with DPBS, fixed in 4% paraformaldehyde in DPBS for 15 min, and permeabilized with 0.1% Triton X-100 in DPBS for 10 min at room temperature, followed by washing with 0.1% gelatin in DPBS. The permeabilized cells were incubated with anti-FLAG antibody (1∶1000) in DPBS for 1 h at room temperature. After washing with 0.1% gelatin in DPBS, the cells were incubated with a fluorescein isothiocyanate-conjugated anti-mouse IgG antibody or an Alexa Fluor 594 rabbit anti-mouse IgG antibody for 1 h at room temperature. After washing with 0.1% gelatin in DPBS, the cells were observed using a Leica AF7000 fluorescence microscope. The quantitative analysis of the ER morphological changes was performed by fluorescence microscopic observation of 100 immunofluorescence-positive (transfected) cells. The extent of ER morphological changes was expressed as a percentage of the number of cells having highly tubular, partially tubular, and non-tubular ER against the total number of transfected cells. Data are expressed as mean ± SD for 5 independent experiments.

### Statistical analysis

Statistical analysis was carried out using two-tailed *t* test (Microsoft Excel; Microsoft). The means of two distributions were considered significantly different if p<0.05.

## Results

### Protein Lunapark, an integral membrane protein, is *N*-myristoylated

**Figure 1:**
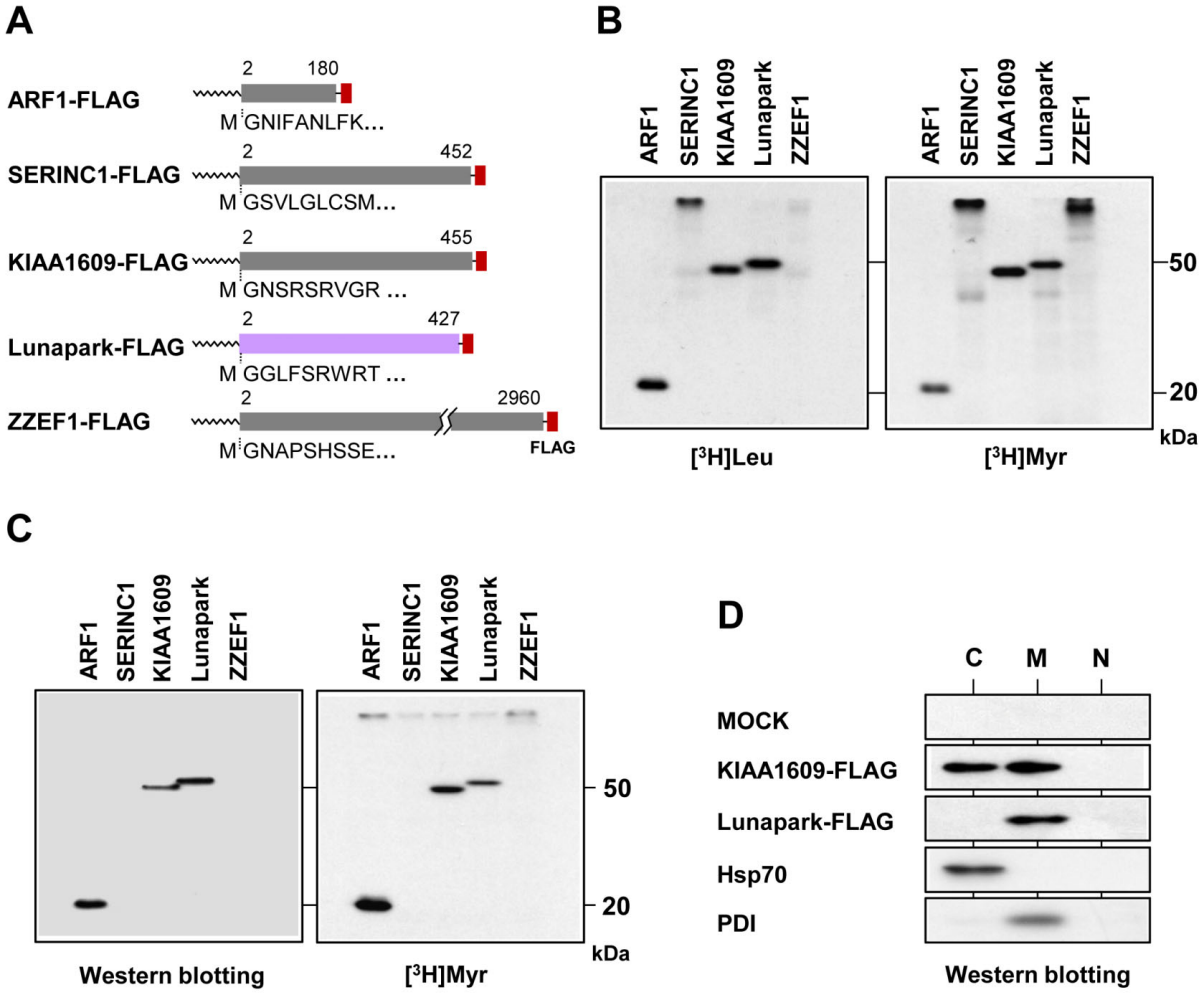
Protein Lunapark is an *N*-myristoylated integral membrane protein. A. Structure and N-terminal sequence of cDNA clones analyzed in this study. B. mRNAs encoding ARF1-, SERINC1-, KIAA1609-, Lunapark- and ZZEF1-FLAG were translated *in vitro* in the presence of [^3^H]leucine ([^3^H]Leu) or [^3^H]myristic acid ([^3^H]Myr) using an insect cell-free protein synthesis system. The labeled translation products were analyzed by SDS-PAGE and fluorography. C. cDNAs encoding ARF1-, SERINC1-, KIAA1609-, Lunapark- and ZZEF1-FLAG were transfected in to COS-1 cells, and their expression and *N*-myristoylation of the products were evaluated by Western blotting analysis and [^3^H]myristic acid labeling, respectively. D. KIAA1609-FLAG and Lunapark-FLAG were expressed in COS-1 cells, and subcellular fractionation experiments were performed. Hsp70 was used as a cytosolic marker protein and PDI was used as membrane/organelle marker protein. C, cytosolic fraction; M, membrane/organelle fraction; N, nuclear fraction.

**Figure 2:**
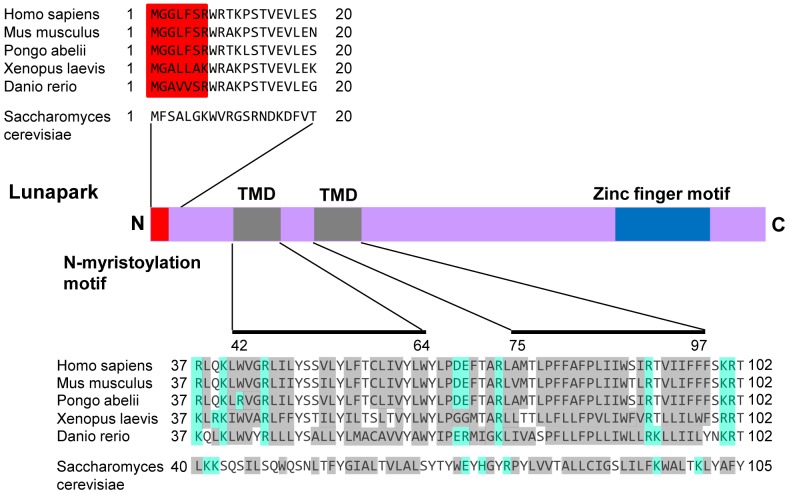
Structure of protein Lunapark. Alignment of the N-terminal sequences and the transmembrane domains (TMDs) of the Lunapark family of proteins is shown. *N*-myristoylation motifs are shown in red in the N-terminal sequence. Hydrophobic amino acids are shown in grey and charged amino acids in blue in the transmembrane domain and their flanking regions. The predicted transmembrane domains are indicated as solid lines.

We first searched for novel human *N*-myristoylated transmembrane proteins using four recently identified human *N*-myristoylated proteins with predicted transmembrane domains. In the previous study, we identified 18 novel human *N*-myristoylated proteins out of approximately 2,000 KOP human cDNA clones by metabolic labeling and mass spectrometric analyses of proteins expressed using an insect cell-free protein synthesis system [30]. Bioinformatic analysis of these proteins using SOSUI, a prediction system for membrane proteins [36], suggested that four out of the 18 novel *N*-myristoylated proteins are integral membrane proteins. Therefore, in this study, the membrane localization and membrane integration of these proteins was examined. The cDNA clones encoding the four *N*-myristoylated proteins (serine incorporator 1 (SERINC1), KIAA1609, protein Lunapark (Lunapark), and ZZEF1) analyzed in this study are listed in Table 1. To confirm that these proteins are *N*-myristoylated, metabolic labeling experiments in an insect cell-free protein synthesis system were performed using mRNAs encoding these proteins with FLAG-tags at their C-termini. In this experiment, FLAG-tagged ARF1 protein was used as positive control. As shown in the left panel of Figure 1B, all the proteins were expressed, as determined by the incorporation of [^3^H]leucine. In this case, the relative mobility in the SDS-PAGE gel of *in vitro* translation products of SERINC1 was very low probably because it contains many transmembrane domains. The results of [^3^H]myristic acid incorporation revealed that all the products were efficiently *N*-myristoylated, as shown in the right panel of Figure 1B. When these cDNAs were transfected to COS-1 cells and metabolic labeling with [^3^H]myristic acid was performed, only two proteins (KIAA1609 and protein Lunapark) were expressed efficiently, as determined by the Western blotting analysis of total cell lysates of the transfected cells (Fig. 1C, left panel). The results of [^3^H]myristic acid incorporation revealed that both of the expressed proteins were efficiently *N*-myristoylated, as shown in the right panel of Figure 1C. To determine the intracellular localization of these two proteins, subcellular fractionation experiments using COS-1 cells expressing these two proteins were performed. As shown in Figure 1D, protein Lunapark was fractionated exclusively in membrane/organelle fraction as was the case with PDI, a membrane/organelle marker protein. In contrast, KIAA1609 fractionated in to both cytosolic and membrane/organelle fractions. These results suggested that KIAA1609 is not an integral transmembrane protein. Therefore, further analyses were performed using protein Lunapark. Protein Lunapark belongs to the Lunapark family of proteins. It has been reported that this protein might be involved in the development of limbs and the central nervous system [29]. However, a recent report revealed that Lnp1p, the yeast ortholog of protein Lunapark, is a membrane protein of the ER and is involved in network formation of the ER [28]. Protein Lunapark has orthologous counterparts in plants, fungi, and animals such as in *Caenorhabditis elegans*, Drosophila, and vertebrates. Interspecies alignments revealed that the N-terminal *N*-myristoylation motif, two putative transmembrane domains, and C-terminal zinc finger motif were highly conserved among the members of this protein family (Fig. 2).

**Table 1:** Characteristics of the Gene Products of the four cDNA Clones.

FXC no.	Protein name	Gene name	Accession no.	Length (aa)	Protein function	No of putative transmembrane domains
FXC00782	Serine incorporator 1	SERINC1	AB384156	453	Serine incorporator	11
FXC00876	TLD domain-containing protein KIAA1609	KIAA1609	AB384249	456	Unknown	1
FXC00896	Protein Lunapark	LNP	AB384269	428	Unknown	2
FXC01083	Zinc finger ZZ-type and EF-hand domain-containing protein 1	ZZEF1	AB384472	2961	Unknown	2

List of cDNA clones analyzed in this study.

### Analysis of protein *N*-myristoylation using *in vitro*-synthesized protein Lunapark

**Figure 3:**
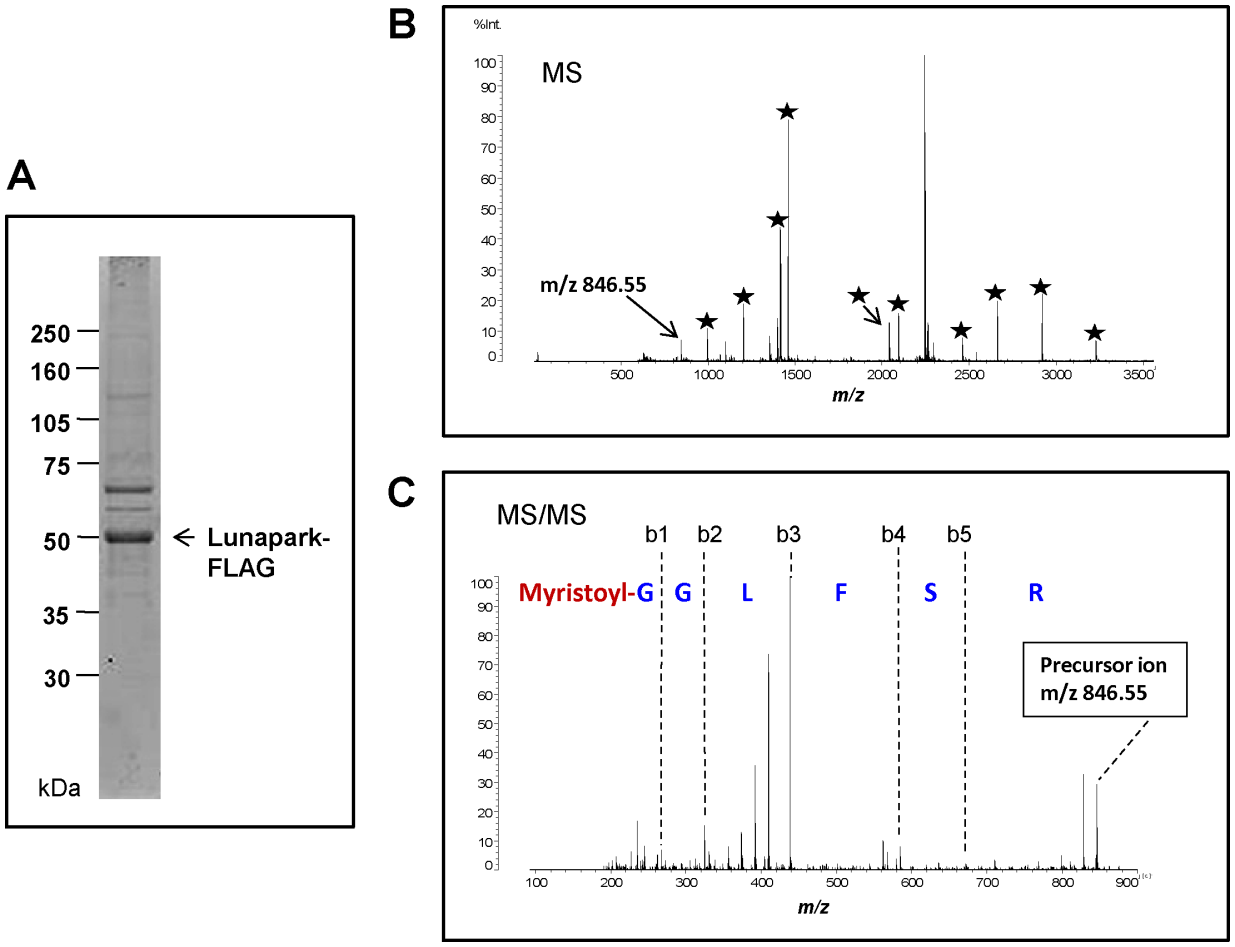
Detection of protein *N*-myristoylation in *in vitro*-synthesized protein Lunapark by MS analysis. A. Purified *in vitro*-synthesized protein Lunapark (1 µg) was separated by electrophoresis in an SDS-PAGE gel (12.5%). B. MALDI-MS of the tryptic peptides from *in vitro-*synthesized protein Lunapark. The peaks of the tryptic peptides derived from *in vitro*-synthesized protein Lunapark are indicated by stars. C. MS/MS analysis was performed for the peak at m/z 846.55. The identified N-myristoylated N-terminal sequence is shown.

To confirm that protein *N*-myristoylation occurs on the N-terminal Gly residue of protein Lunapark, mass spectrometric analyses were performed on *in vitro*-synthesized protein Lunapark. *In vitro*-synthesized and affinity-purified protein Lunapark was detected as a main band with an apparent molecular mass of 49 kDa by SDS-PAGE analysis (Fig. 3A). The protein band was reduced, S-alkylated, and then digested with trypsin. The tryptic digests were analyzed by MALDI-TOF MS. The MS spectrum of this sample revealed that most of the detected tryptic peptides were derived from protein Lunapark (Fig. 3B). A peak equivalent to the *N-*myristoylated tryptic peptide was observed at m/z 846.55 (Fig. 3B). This peak was identified as the *N-*myristoylated tryptic fragment of protein Lunapark, *N-*myristoyl-Gly-Gly-Leu-Phe-Ser-Arg, by MS/MS analysis (Fig. 3C). These results clearly indicated that the protein *N*-myristoylation occurs on the N-terminal Gly residue of protein Lunapark.

### Analysis of membrane integration and membrane topology of protein Lunapark

**Figure 4:**
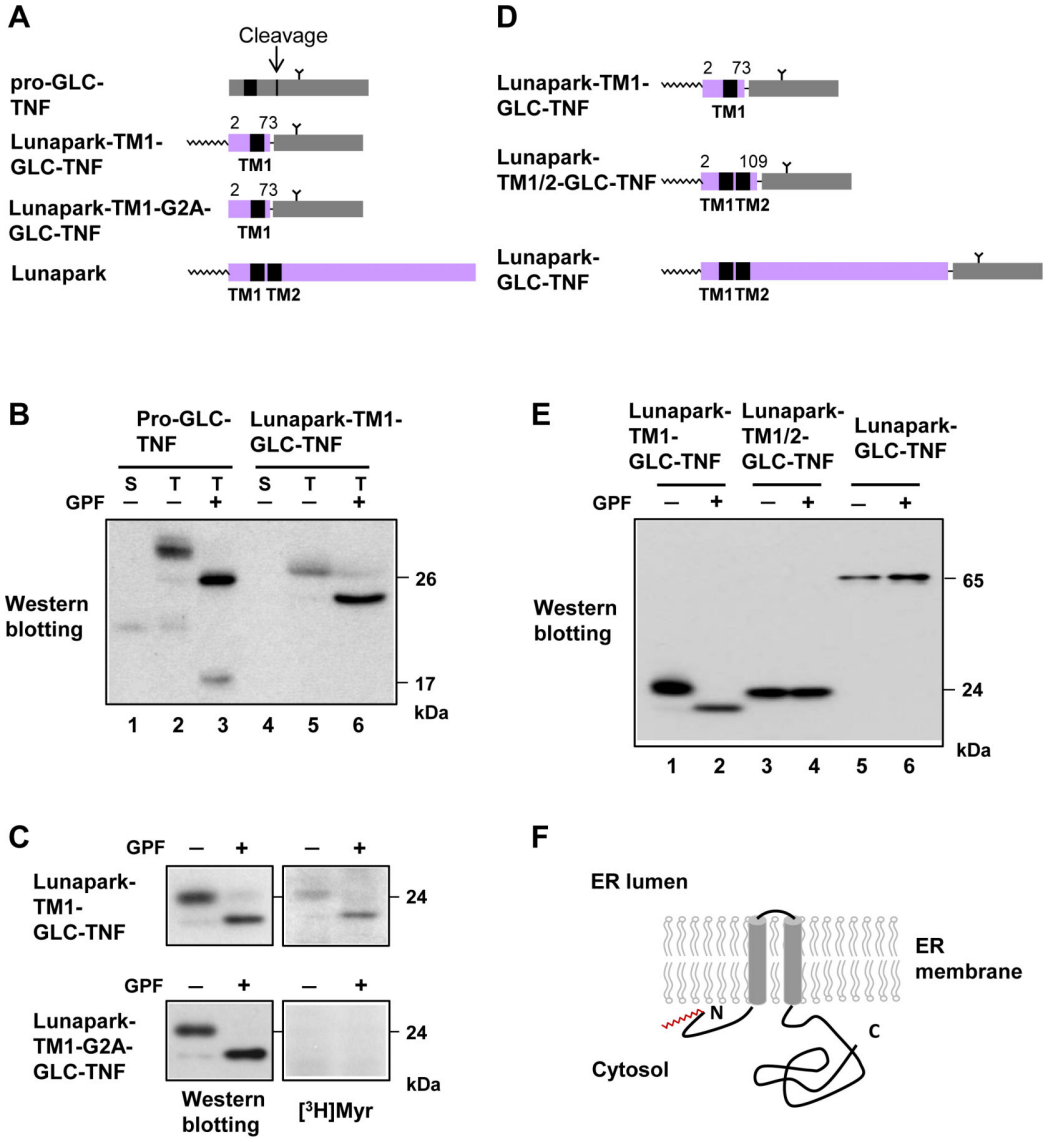
Analysis of membrane integration and membrane topology of protein Lunapark. A. Structure of pro-GLC-TNF, Lunapark-TM1-GLC-TNF, and Lunapark-TM1-G2A-GLC-TNF for analysis of the function of transmembrane domain 1 (TM1) of protein Lunapark. B. cDNAs encoding pro-GLC-TNF, Lunapark-TM1-GLC-TNF were transfected in to COS-1 cells, and their secretion and expression in total cell lysates were evaluated by Western blotting analysis using an anti-TNF antibody. *N*-glycosylation of the expressed proteins in total cell lysates was determined by the change in its molecular weight after treatment with glycopeptidase F (GPF). S, cell culture supernatant; T, total cell lysates. C. cDNAs encoding Lunapark-TM1-GLC-TNF and Lunapark-TM1-G2A-GLC-TNF were transfected in to COS-1 cells, and their expression and the *N*-myristoylation of the products in total cell lysates were evaluated by Western blotting analysis and [^3^H]myristic acid ([^3^H]Myr) labeling, respectively. *N*-glycosylation of the expressed protein in total cell lysates was determined by the change in molecular weight after treatment with GPF. D. Structure of Lunapark-TM1-GLC-TNF, Lunapark-TM1/2-GLC-TNF, and Lunapark-GLC-TNF for analysis of the function of transmembrane domain 2 (TM2) of protein Lunapark. E. cDNAs encoding Lunapark-TM1-GLC-TNF, Lunapark-TM1/2-GLC-TNF, and Lunapark-GLC-TNF were transfected in to COS-1 cells, and their expression in total cell lysates was evaluated by Western blotting analysis using an anti-TNF antibody. *N*-glycosylation of the expressed proteins in total cell lysates was determined by the change in molecular weight after treatment with GPF. F. Schematic representation of the transmembrane topology of protein Lunapark in the ER membrane.

Since biochemical characterization of membrane integration and membrane topology of protein Lunapark has not been performed, the role of the two putative transmembrane domains and their flanking regions of protein Lunapark on membrane translocation and membrane topology formation was evaluated using TNF-fusion proteins with each of these domains. When pro-GLC-TNF, a model *N*-glycosylated type II transmembrane protein (single spanning membrane protein with N-cyto/C-exo topology) with an *N*-glycosylation site in the mature domain of TNF, was expressed in COS-1 cells, a protein band with a molecular mass of 30 kDa was detected in the total cell lysates, as shown in Figure 4B, lane 2. Glycopeptidase F (GPF) treatment of this protein gave rise to a protein band with an approximately 4 kDa smaller molecular mass (26 kDa), indicating that pro-GLC-TNF is *N*-glycosylated when expressed in HEK293T cells (Fig. 4B, lane 3). In this case, 20 kDa TNF was detected in the cell culture supernatant (Fig. 4B, lane 1), suggesting that pro-GLC-TNF was cleaved at the cell surface to generate the secreted form of *N*-glycosylated TNF. When the same experiment was performed using Lunapark-TM1-GLC-TNF, in which the mature domain of pro-GLC-TNF was fused to the C-terminus of the N-terminal 73 residues of protein Lunapark, including putative TM1 and its flanking regions, a similar reduction in the molecular weight of the protein was observed after GPF treatment (Fig. 4B, lanes 5 and 6). The absence of a protein band in the cell culture supernatant indicated that cleavage of Lunapark-TM1-GLC-TNF at the cell surface did not occur (Fig. 4B, lane 4). These results clearly indicated that TM1 and its flanking regions of protein Lunapark functioned as a type II signal anchor sequence and generated single spanning membrane protein with type II (N-cyto/C-exo) topology. When COS-1 cells transfected with Lunapark-TM1-GLC-TNF cDNA were labeled with [^3^H]myristic acid and then the same experiment was performed, protein *N*-myristoylation was observed both on *N*-glycosylated and de-glycosylated protein bands (Fig. 4C, upper panels), indicating that protein *N*-myristoylation did occur on the integral transmembrane protein.

To evaluate the role of TM2 and its flanking regions on the membrane topology formation of protein Lunapark, the same experiment was performed using Lunapark-TM1/2-GLC-TNF in which the mature domain of pro-GLC-TNF was fused to the C-terminus of the N-terminal 109 residues of protein Lunapark including putative TM1 and TM2 and its flanking regions. As shown in Figure 4E, lanes 3 and 4, GPF treatment did not affect the molecular weight of the protein, indicating that TM2 and its flanking region functioned as a stop-transfer sequence and blocked the membrane translocation of the mature domain of pro-GLC-TNF. When the mature domain of pro-GLC-TNF was fused to the C-terminus of the full-length of protein Lunapark, a similar pattern of protein bands as with that of Lunapark-TM1/2-GLC-TNF was observed after GPF treatment (Fig. 4E, lanes 5 and 6). These results suggested that membrane translocation and topology formation of protein Lunapark was mediated by the two transmembrane domains, TM1 and TM2 and their flanking regions, and no other regions were involved in the membrane translocation of this protein.

To confirm the function of TM2 and its flanking regions of protein Lunapark as a stop-transfer sequence, the ability to block membrane translocation was evaluated by testing the ability of this region to block the secretion of a secretory protein. For this purpose, H14-TNF-Lunapark-TM2, in which TM2 and its flanking regions of protein Lunapark were fused to the C-terminus of H14-TNF, a model secretory protein, was generated. When the H14-TNF cDNA was transfected to COS-1 cells, mature secretory TNF was detected in the cell-culture supernatant, as shown in Figure S1B, lane 1. In the case of H14-TNF-Lunapark-TM2, the secretion of mature TNF was completely inhibited (Fig. S1B, lane 3). When TM2 and its C-terminal flanking region (Ala74-Glu107) was deleted from this construct, efficient secretion of mature TNF was observed, as shown in Figure S1B, lane 5. These results clearly indicated that TM2 of protein Lunapark functioned as a stop-transfer sequence and blocked the secretion of mature TNF from the cells.

### Protein *N*-myristoylation is not required for the membrane translocation of protein Lunapark

To determine whether protein *N*-myristoylation is required for the membrane translocation of protein Lunapark mediated by TM1 and its flanking regions, membrane translocation of Lunapark-TM1-G2A-GLC-TNF, in which the *N*-myristoylation motif is disrupted by replacing Gly2 with Ala, was evaluated. As shown in Figure 4C left panels, the molecular weight of expressed Lunapark-TM1-G2A-GLC-TNF was similar to that of Lunapark-TM1-GLC-TNF. In addition, a similar reduction of molecular weight of protein was observed after GPF treatment. As shown in Figure 4C, lower right panel, [^3^H]myristic acid incorporation was not observed on *N*-glycosylated and de-glycosylated protein bands of Lunapark-TM1-G2A-GLC-TNF. These results indicated that membrane translocation mediated by TM1 and its flanking regions proceeds irrespective of protein *N*-myristoylation. Thus, protein *N*-myristoylation occurs on protein Lunapark is not required for membrane translocation and membrane topology formation of protein Lunapark.

### ER morphological changes induced by overexpression of protein Lunapark and its dependency on protein *N*-myristoylation

**Figure 5:**
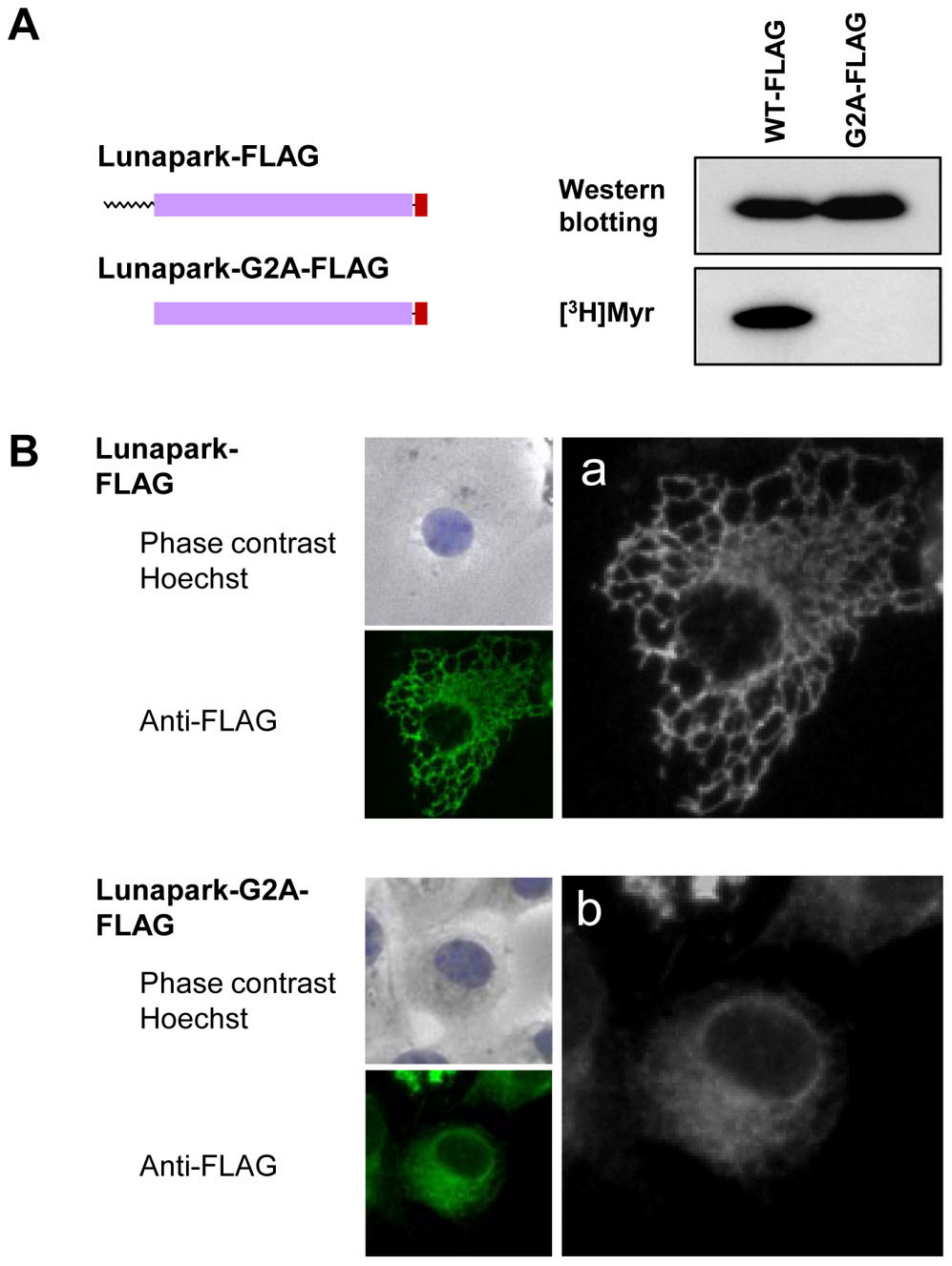
Protein *N*-myristoylation plays a critical role in the ER morphological change induced by protein Lunapark. A. Detection of protein *N*-myristoylation of Lunapark-FLAG expressed in HEK293T cells. cDNAs encoding Lunapark-FLAG and Lunapark-G2A-FLAG were transfected in to HEK293T cells, and their expression and the *N*-myristoylation of the products in total cell lysates were evaluated by Western blotting analysis and [^3^H]myristic acid ([^3^H]Myr) labeling, respectively. B. Intracellular localization of Lunapark-FLAG and Lunapark-G2A-FLAG was determined by immunofluorescence staining of HEK293T cells transfected with cDNAs encoding these two proteins using an anti-FLAG antibody. a and b show a close-up view of the immunofluorescence image.

**Figure 6:**
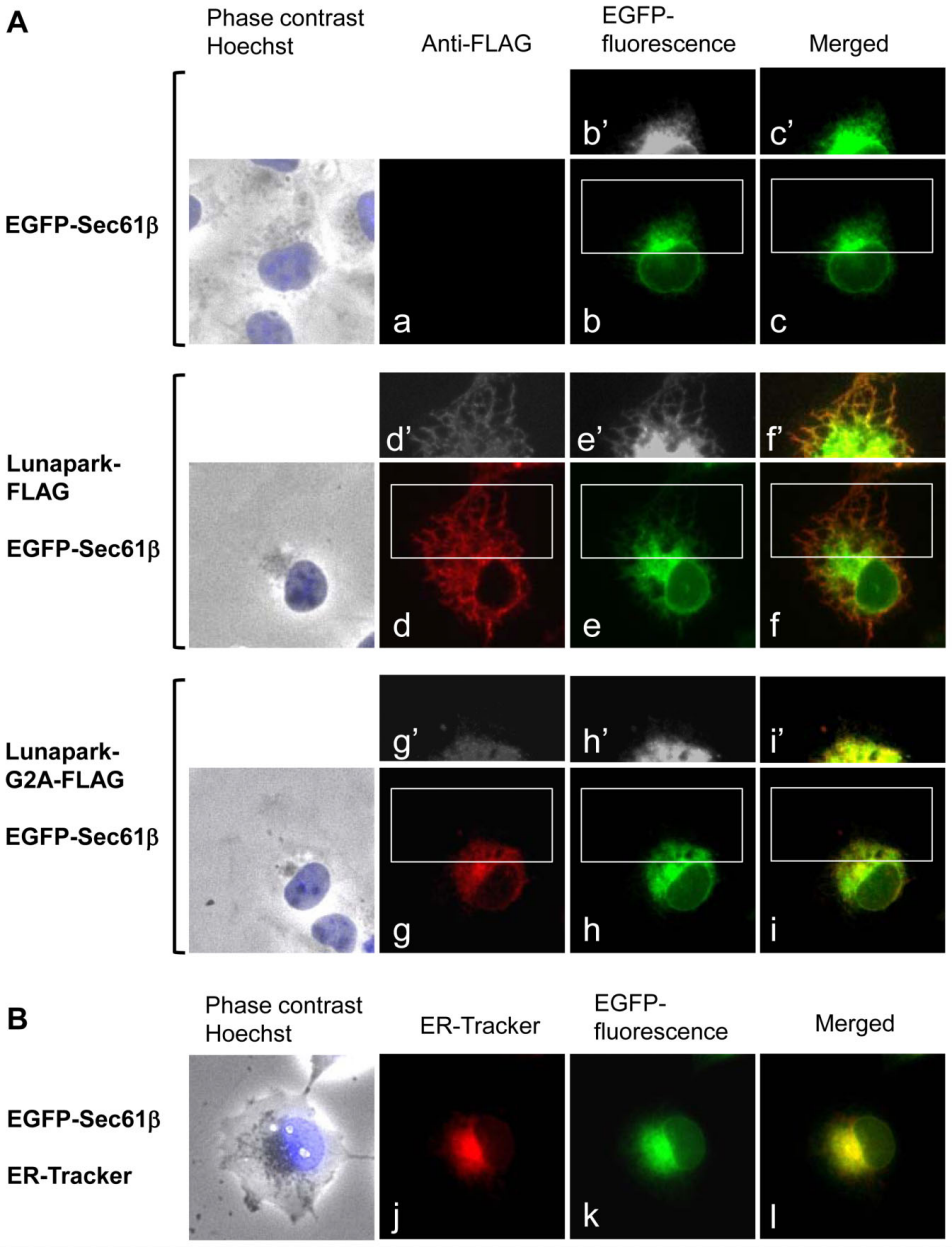
Protein Lunapark mainly localized to the peripheral ER and induced the ER morphological change in an *N*-myristoylation-dependent manner. A. EGFP-Sec61β cDNA alone, Lunapark-FLAG cDNA and EGFP-Sec61β cDNA, or Lunapark-G2A-FLAG cDNA and EGFP-Sec61β cDNA, were transfected in to HEK-293T cells, and distribution of these proteins was evaluated by immunofluorescence analysis or fluorescence microscopic analysis. b′, c′, d′, e′, f′, g′, h′, and i′ show a close-up and over-exposed image of the area surrounded by a white box in b, c, d, e, f, g, h, and i, respectively. B. EGFP-Sec61β cDNA was transfected in to HEK-293T cells and the distribution of the protein was evaluated by fluorescence microscopic analysis. ER was detected with ER-Tracker Red.

In order to determine the role of protein *N*-myristoylation of protein Lunapark in the intracellular localization of this protein, cDNAs encoding protein Lunapark C-terminally tagged with FLAG tag and its G2A mutant were generated, and their intracellular localization was determined by immunofluorescence analysis of HEK293 T cells transfected with these cDNAs. As shown in Figure 5A, [^3^H]myristic acid labeling revealed that Lunapark-FLAG was efficiently *N*-myristoylated, whereas incorporation of [^3^H]myristic acid was not observed on Lunapark-G2A-FLAG. Immunofluorescence analysis of HEK293T cells transfected with cDNA encoding Lunapark-FLAG revealed that overexpressed Lunapark-FLAG showed a characteristic distribution pattern with large polygonal tubular structures, as shown in Figure 5B, a. In contrast, in the case of Lunapark-G2A-FLAG, the characteristic distribution pattern with a large polygonal tubular structure was not observed, as shown in Figure 5B, b. Because a reticular network structure was observed on the ER, it was speculated from these observations that protein Lunapark specifically localized to the ER and induced tubular ER formation in an *N*-myristoylation-dependent manner. In order to prove this, effect of overexpression of protein Lunapark on the distribution of ER localized protein was evaluated. For this experiment, EGFP-Sec61β was used as general ER marker protein that distributes throughout the ER [37]. In HEK293T cells expressing EGFP-Sec61β, perinuclear localization of EGFP-Sec61β was observed as shown in Figure 6A, b. In this case, peripheral tubular structure was not observed even in the overexposed black-and-white image (b′). When HEK293T cells expressing EGFP-Sec61β were stained with ER-Tracker Red, EGFP-fluorescence of EGFP-Sec61β completely merged with fluorescence of ER-Tracker Red as shown in Figure 6B, j,k,l. These results clearly demonstrated the specific localization of EGFP-Sec61β to the ER. When EGFP-Sec61β was coexpressed with Lunapark-FLAG, large polygonal structure around the perinuclear sheet was observed with EGFP-Sec61β (e,e′). This peripheral tubular structure detected with EGFP-Sec61β was well merged with the peripheral tubular structure observed with Lunapark-Flag (d′,e′,f′). In contrast, when EGFP-Sec61β was coexpressed with non-myristoylated Lunapark-G2A-FLAG, the characteristic distribution pattern with a large polygonal tubular structure was not observed with EGFP-Sec61β, as is the case with the distribution pattern of Lunapark-G2A-FLAG, as shown in Figure 6A, g,g′,h,h′. These results suggested that protein Lunapark induced tubular ER formation in an *N*-myristoylation-dependent manner. In order to further confirm this, quantitative analysis of the morphological change of the ER in cells expressing both EGFP-Sec61β and protein Lunapark (EGFP-positive/immunofluorescence-positive cells) was performed. As shown in Figure S2A and B, significant difference in the induction of the ER morphological change was observed between Lunapark-FLAG and Lunapark-G2A-FLAG, and highly tubular ER was observed only on cells expressing wild-type Lunapark-FLAG. These results clearly indicated that protein Lunapark localized to the peripheral ER and induced tubular ER formation in an *N*-myristoylation-dependent manner.

### Both protein *N*-myristoylation and the C-terminal zinc-finger domain of protein Lunapark contribute to the ER morphological change induced by protein Lunapark

**Figure 7:**
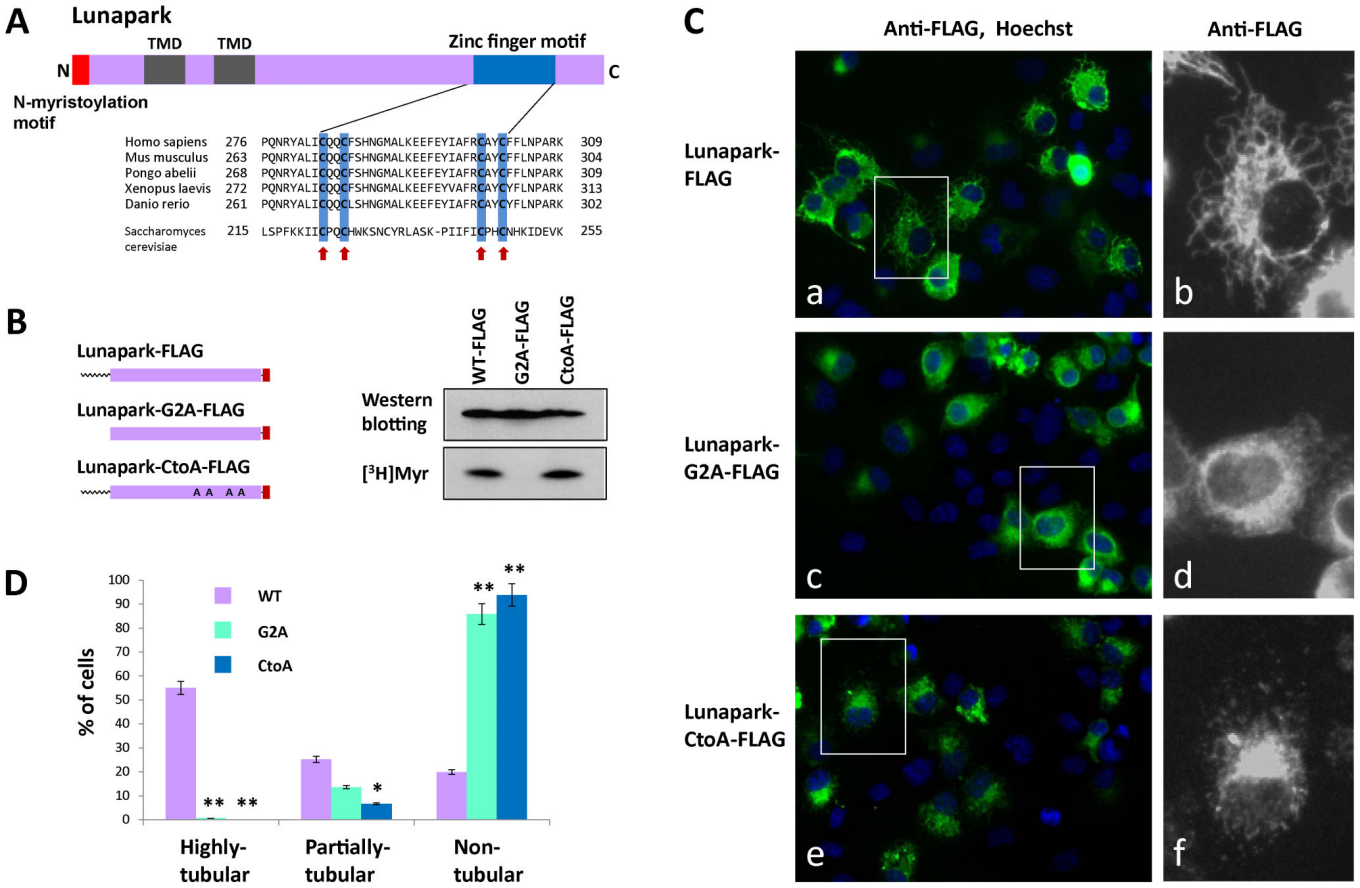
Role of the zinc finger motif of protein Lunapark in the ER morphological change induced by protein Lunapark. A. Alignment of the zinc finger motif of Lunapark protein family members. Highly conserved cysteine residues are indicated by red arrows. B. Detection of protein *N*-myristoylation of Lunapark-CtoA-FLAG expressed in HEK293T cells. cDNAs encoding Lunapark-FLAG, Lunapark-G2A-FLAG, and Lunapark-CtoA-FLAG were transfected in to HEK293T cells, and their expression and the *N*-myristoylation of the products in the total cell lysates were evaluated by Western blotting analysis and [^3^H]myristic acid ([^3^H]Myr) labeling, respectively. C. Intracellular localization of Lunapark-FLAG, Lunapark-G2A-FLAG, and Lunapark-CtoA-FLAG was determined by immunofluorescence staining of HEK293T cells transfected with cDNAs encoding these three proteins using an anti-FLAG antibody. Right panel shows a close-up view of the area surrounded by a white box in the immunofluorescence image. D. Quantitative analysis of the ER morphological change in HEK293T cells induced by Lunapark-FLAG (WT), Lunapark-G2A-FLAG (G2A), and Lunapark-CtoA-FLAG (CtoA). cDNAs encoding Lunapark-FLAG, Lunapark-G2A-FLAG, and Lunapark-CtoA-FLAG were transfected in to HEK293T cells and the morphological change of the ER in each cell was determined by immunofluorescence staining and the extent of the ER morphological change was compared. The extent of ER morphological changes is expressed as a percentage of the number of cells with highly tubular, partially tubular, and non-tubular ER against the total number of transfected cells. Data are expressed as mean ± SD for five independent experiments. **P<0.001 vs. WT. *P<0.01 vs. WT.

In addition to the N-terminal *N*-myristoylation motif and two transmembrane domains, the C-terminal zinc finger motif was highly conserved among the members of the Lunapark family of proteins, as shown in Figure 7A. To determine the functional role of the C-terminal zinc finger motif, a mutant (Lunapark-CtoA-FLAG), in which the four conserved cysteine residues in the zinc finger motif (Fig. 7A, arrows) were mutated to alanine, was generated and its ability to induce ER morphological changes was evaluated by immunofluorescence microscopy. [^3^H]myristic acid labeling of HEK293T cells transfected with cDNA encoding Lunapark-CtoA-FLAG revealed that this protein is *N*-myristoylated efficiently as observed with wild-type Lunapark-FLAG (Fig. 7B). In this experiment, the level of protein expression of these three proteins was similar, as determined by the Western blotting analysis. As shown in Fig. 7C, a,b, transfection of HEK293T cells with the cDNA encoding Lunapark-FLAG induced the formation of the large polygonal tubular structure of ER. In contrast, in the case of HEK293T cells transfected with a cDNA encoding Lunapark-CtoA-FLAG, the characteristic tubular ER formation was not observed (Fig. 7C, e,f), as was the case with a cDNA encoding Lunapark-G2A-FLAG (Fig. 7C, c,d). In order to further confirm the role of protein *N*-myristoylation and the C-terminal zinc finger motif on the ER morphological change induced by protein Lunapark, quantitative analysis of the morphological change of the ER in cells transfected with these three cDNAs was performed. For this experiment, the extent of ER morphological change was compared using immunofluorescence images of cells transfected with these three cDNAs. As a result, significant differences in the induction of the ER morphological change were observed among these three proteins, as shown in Figure 7D. In the case of Lunapark-FLAG, peripheral tubular structures (highly tubular plus partially tubular) were observed in approximately 80% of the transfected (immunofluorescence-positive) cells. In contrast, as for Lunapark-G2A-FLAG and Lunapark-CtoA-FLAG, peripheral tubular structures were not observed on 80–90% of the transfected cells and only partially tubular structures were observed in 10–20% of the transfected cells (Fig. 7D). These results clearly indicated that both protein *N*-myristoylation and the C-terminal zinc finger domain play critical roles in the ER morphological change induced by protein Lunapark.

## Discussion

Protein *N*-myristoylation occurs mainly on cytoplasmic proteins in eukaryotic cellular proteins and only very few integral membrane proteins have been demonstrated to be *N*-myristoylated so far. In the present study, we searched for human *N*-myristoylated transmembrane proteins and studied the role of protein *N*-myristoylation on membrane translocation, membrane topology formation, intracellular targeting, and physiological function of the proteins. Recently, we have established a strategy for the comprehensive identification of human *N-*myristoylated proteins from human cDNA clones in human cDNA resources by metabolic labeling and mass spectrometric analyses of proteins expressed using an insect cell-free protein synthesis system [30], [38]. Using this strategy we have identified 27 *N*-myristoylated proteins out of approximately 2,000 Kazusa ORFeome project (KOP) human cDNA clones. Database searches with these 27 cDNA clones revealed that 18 encoded novel *N-*myristoylated proteins. Bioinformatic analysis of these proteins using SOSUI, a prediction system for membrane proteins [36], suggested that four out of the 18 novel *N*-myristoylated proteins are integral membrane proteins. Therefore, in the present study, membrane localization and membrane integration of these four proteins were studied. As a result, it was found that protein Lunapark, the human homologue of yeast protein Lnp1p [28], which has recently been found to be involved in network formation of the ER, is an *N*-myristoylated double-spanning integral membrane protein of ER with an N-/C-terminal cytoplasmic orientation (Fig. 1,3,4). As previously described, it has been reported that both of two mammalian *N*-myristoylated transmembrane proteins so far discovered (b5R and DES1) are dually targeted to the ER and mitochondria, and protein N-myristoylation favors to localization of these two proteins to the mitochondria [23], [26]. These observations might indicate that protein *N*-myristoylation functions as a mitochondrial targeting signal in mammalian integral transmembrane proteins. However, the present study clearly demonstrated that *N*-myristoylated protein Lunapark localizes exclusively to the ER. Thus, protein *N*-myristoylation does not necessarily function as a mitochondrial targeting signal of mammalian integral transmembrane proteins. The *N*-myristoylation occurring on protein Lunapark is not required for membrane translocation or topology formation of protein Lunapark (Fig. 4). Instead, it was revealed that protein *N*-myristoylation plays critical role in the ER morphological change induced by overexpression of protein Lunapark (Fig. 5, 6, 7).

The ER plays a critical role in many cellular processes, such as protein synthesis, lipid synthesis, protein modification and the regulation of Ca^2+^ homeostasis [39]. It consists of an extended polygonal network of tubules connecting to ER sheets and the nuclear envelope. The ER network extends throughout the cell by a dynamic process of tubule extension and tubule fusion [40], [41]. Several protein families are involved in the generation and maintenance of the distinctive architecture of ER. The reticulons and DP1/Yop1p play central roles in the generation and maintenance of the reticular structure of ER [42], [43]. Atlastin, a dynamin-like GTPase, promotes ER tubule fusion, which leads to the formation of new three-way junctions within the polygonal network [44], [45]. In addition to these proteins, it was reported recently that a yeast protein Lnp1p, a member of the Lunapark family of proteins, is required for ER network formation [28]. Lnp1p was found to interact with the reticulons, Yop1p, and Sey1p (yeast ortholog of atlastin) and localize to the ER tubule junctions in both yeast and mammalian cells. It was also found that the interaction of Lnp1p with reticulon and the localization of Lnp1p to ER junctions are regulated by Sey1p. From these results, it was proposed that Lnp1p and Sey1p act antagonistically to balance polygonal network formation.

In the present study, we showed that protein Lunapark, the human ortholog of Lnp1p, is *N*-myristoylated and localized mainly to the peripheral ER and induced the formation of large polygonal tubular structures when overexpressed in human cells. The morphological change in the ER induced by protein Lunapark was significantly inhibited by the inhibition of protein *N*-myristoylation by the replacement of Gly2 with Ala, suggesting that protein *N*-myristoylation plays a critical role in the ER morphological change induced by overexpression of protein Lunapark. As shown in Figure 2 and 7A, the N-terminal *N*-myristoylation motif, two transmembrane domains, and the C-terminal zinc finger domain were highly conserved among the Lunapark family of proteins. Interestingly, however, the *N*-myristoylation motif was not found in yeast Lnp1p. In addition, the amino acid sequences of N-terminal region, two transmembrane domains, and the C-terminal zinc finger domain of yeast Lnp1p are significantly different from those of other Lunapark family members. In fact, the peptide LNPARK, which is present near the C-terminus of the zinc finger domain of most of the Lunapark family of proteins and is the origin of the name Lunapark [29], is not present in Lnp1p. Thus, the amino acid sequence, the susceptibility to protein *N*-myristoylation, and the intracellular localization of human protein Lunapark were different from those of yeast protein Lnp1p. From these observations, it is speculated that the structure and the physiological function of yeast Lnp1p might be different from those of other Lunapark family members. The molecular mechanism by which protein *N*-myristoylation strongly affects the ER morphological change induced by protein Lunapark is not clear. In general, the functions of *N*-myristoylated proteins are regulated by reversible membrane binding mediated by protein *N*-myristoylation [1], [5], [46]. Because protein Lunapark is an integral membrane protein, it is unlikely that protein *N*-myristoylation of protein Lunapark functions as a membrane anchor. In fact, we have shown in this study that neither the ER membrane translocation nor the specific localization to ER membrane required protein *N*-myristoylation. In some *N*-myristoylated proteins, such as CAP-23/NAP-22, specific protein–protein interactions mediated by protein *N*-myristoylation play critical roles in the expression of protein function [47]. Therefore, it is possible that *N*-myristoylation dependent protein–protein interaction might be involved in the function of protein Lunapark. The fact that the *N*-myristoylated Lunapark-CtoA-FLAG, in which the four conserved cysteine residues in the zinc finger motif were mutated to alanine, could not induce the characteristic tubular ER formation (Fig. 7C, e, f), suggested that *N*-myristoylation by itself is not sufficient to induce the ER morphological change. Zinc finger motifs are relatively small protein motifs that fold around one or more zinc ions and are widely found in eukaryotes. Many zinc finger proteins are DNA-binding proteins involved in the regulation of gene expression [48]. In addition to their role as a DNA-binding motif, zinc finger motifs have been shown to mediate protein–protein or protein–lipid interactions [49], [50]. Therefore, it is probable that specific protein–protein or protein–lipid interactions mediated by the zinc finger motif of protein Lunapark plays a critical role in the induction of the ER morphological change. Because yeast Lnp1p was found to interact with the reticulons, Yop1p, and Sey1p [28], human protein Lunapark might also interact with reticulons, DP1, and atlastin. In order to clarify the molecular mechanism of the ER morphological change induced by protein Lunapark, it is necessary to study the interaction of protein Lunapark with these proteins and the role of protein *N*-myristoylation and the zinc finger domain of protein Lunapark on these interactions. Thus, further studies will be required to elucidate the roles of protein *N*-myristoylation on the functions of protein Lunapark or other members of the Lunapark protein family.

In this study, we showed that protein Lunapark, a double-spanning integral membrane protein of the ER, is *N*-myristoylated, and the *N-*myristoylation of protein Lunapark strongly affected the ER morphological change induced by this protein. To the best of our knowledge, this is the first report clarifying the role of protein *N*-myristoylation on the membrane translocation, membrane topology formation, intracellular targeting, and function of a human *N*-myristoylated polytopic membrane protein. The number of human proteins with an N-terminal Met-Gly sequence in all the human proteins listed in the Swiss-Prot protein knowledgebase (∼20,000 proteins) is approximately 1,600. Bioinformatic prediction of protein *N*-myristoylation coupled with a prediction system for membrane proteins predicted that more than 50 proteins with an N-terminal Met-Gly sequence are *N*-myristoylated integral transmembrane proteins. We are currently studying the susceptibility to protein *N*-myristoylation, membrane integration, and physiological role of protein *N*-myristoylation of these candidate proteins.

## Supporting Information

Figure S1 **Analysis of the role of transmembrane domain 2 and its flanking region of protein Lunapark on the membrane topology formation of protein Lunapark.** A. Structure of H14-TNF, H14-TNF-Lunapark-TM2, and H14-TNF-Lunapark-ΔTM2 to analyze the function of transmembrane domain 2 (TM2) of protein Lunapark. B. cDNAs encoding H14-TNF, H14-TNF-Lunapark-TM2, and H14-TNF-Lunapark-ΔTM2 were transfected in to COS-1 cells, and their secretion and expression in total cell lysates were evaluated by Western blotting analysis using an anti-TNF antibody. S, cell culture supernatant; T, total cell lysate.
(TIF)

Figure S2 **Quantitative analysis of the ER morphological change in HEK293T cells induced by Lunapark-FLAG (WT) and Lunapark-G2A-FLAG (G2A).** HEK293T cells were cotransfected with EGFP-Sec61β and Lunapark-FLAG (or Lunapark-G2A-FLAG), and quantitative analysis of the ER morphological change was performed by fluorescence microscopic observation of 100 cells expressing both EGFP-Sec61β and protein Lunapark (EGFP-positive/immunofluorescence-positive cells). A. Left panels (a, d) show merged image of EGFP fluorescence, immunofluorescence, and Hoechst staining. Right panels (b, c, e, f) show a close-up and over-exposed image of the area surrounded by a white box in the left panels (a, d). B. The extent of ER morphological changes is expressed as a percentage of the number of cells having highly tubular, partially tubular, and non-tubular image of EGFP-Sec61β against the total number of EGFP-positive/immunofluorescence-positive cells. Data are expressed as mean ± SD for four independent experiments. **P<0.001 vs. WT.
(TIF)

Table S1 The nucleotide sequences of oligonucleotides used in this study.
(TIF)

Table S2 The strategies for construction of plasmids used in this study.
(TIF)
