# Supplementary figures and images for: Protein N-Myristoylation Plays a Critical Role in the Endoplasmic Reticulum Morphological Change Induced by Overexpression of Protein Lunapark, an Integral Membrane Protein of the Endoplasmic Reticulum
Source: PLoS One. 2013 Nov 4;8(11):e78235. doi: 10.1371/journal.pone.0078235 (PMC3817238; doi:10.1371/journal.pone.0078235)

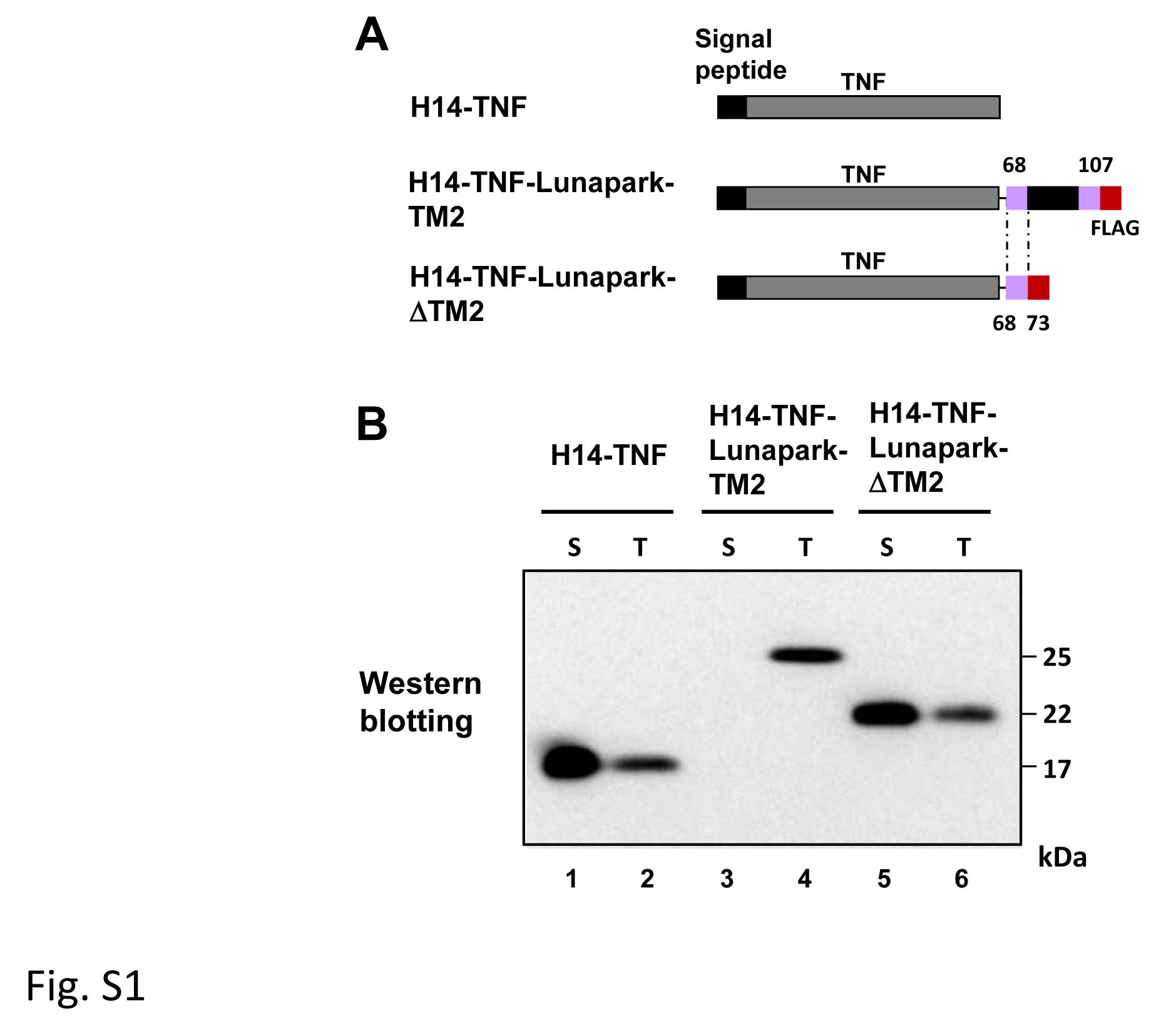

Supplement: Figure S1 — Analysis of the role of transmembrane domain 2 and its flanking region of protein Lunapark on the membrane topology formation of protein Lunapark. A. Structure of H14-TNF, H14-TNF-Lunapark-TM2, and H14-TNF-Lunapark-ΔTM2 to analyze the function of transmembrane domain 2 (TM2) of protein Lunapark. B. cDNAs encoding H14-TNF, H14-TNF-Lunapark-TM2, and H14-TNF-Lunapark-ΔTM2 were transfected in to COS-1 cells, and their secretion and expression in total cell lysates were evaluated by Western blotting analysis using an anti-TNF antibody. S, cell culture supernatant; T, total cell lysate. (TIF) [file pone.0078235.s001.tif]

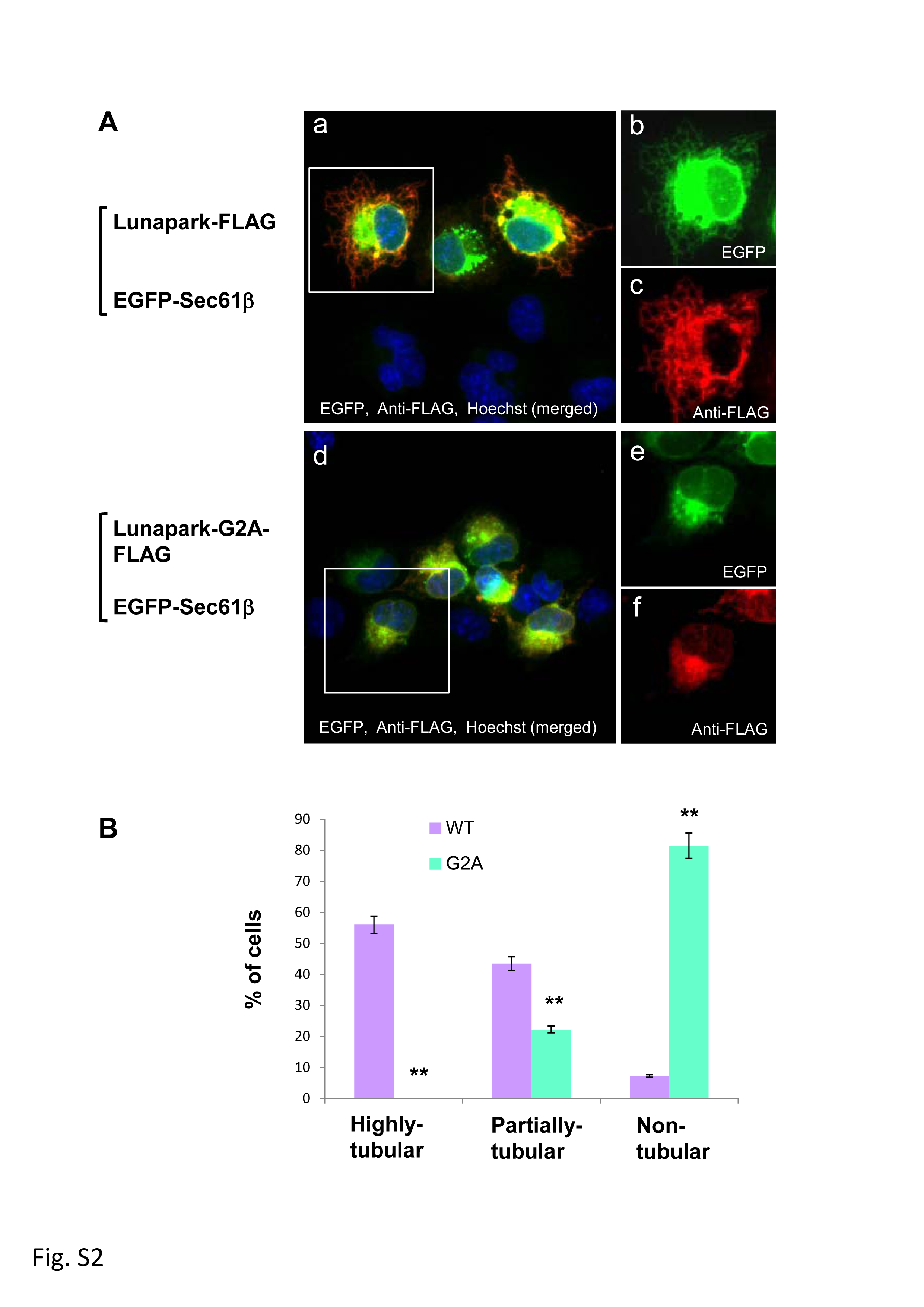

Supplement: Figure S2 — Quantitative analysis of the ER morphological change in HEK293T cells induced by Lunapark-FLAG (WT) and Lunapark-G2A-FLAG (G2A). HEK293T cells were cotransfected with EGFP-Sec61β and Lunapark-FLAG (or Lunapark-G2A-FLAG), and quantitative analysis of the ER morphological change was performed by fluorescence microscopic observation of 100 cells expressing both EGFP-Sec61β and protein Lunapark (EGFP-positive/immunofluorescence-positive cells). A. Left panels (a, d) show merged image of EGFP fluorescence, immunofluorescence, and Hoechst staining. Right panels (b, c, e, f) show a close-up and over-exposed image of the area surrounded by a white box in the left panels (a, d). B. The extent of ER morphological changes is expressed as a percentage of the number of cells having highly tubular, partially tubular, and non-tubular image of EGFP-Sec61β against the total number of EGFP-positive/immunofluorescence-positive cells. Data are expressed as mean ± SD for four independent experiments. **P<0.001 vs. WT. (TIF) [file pone.0078235.s002.tif]

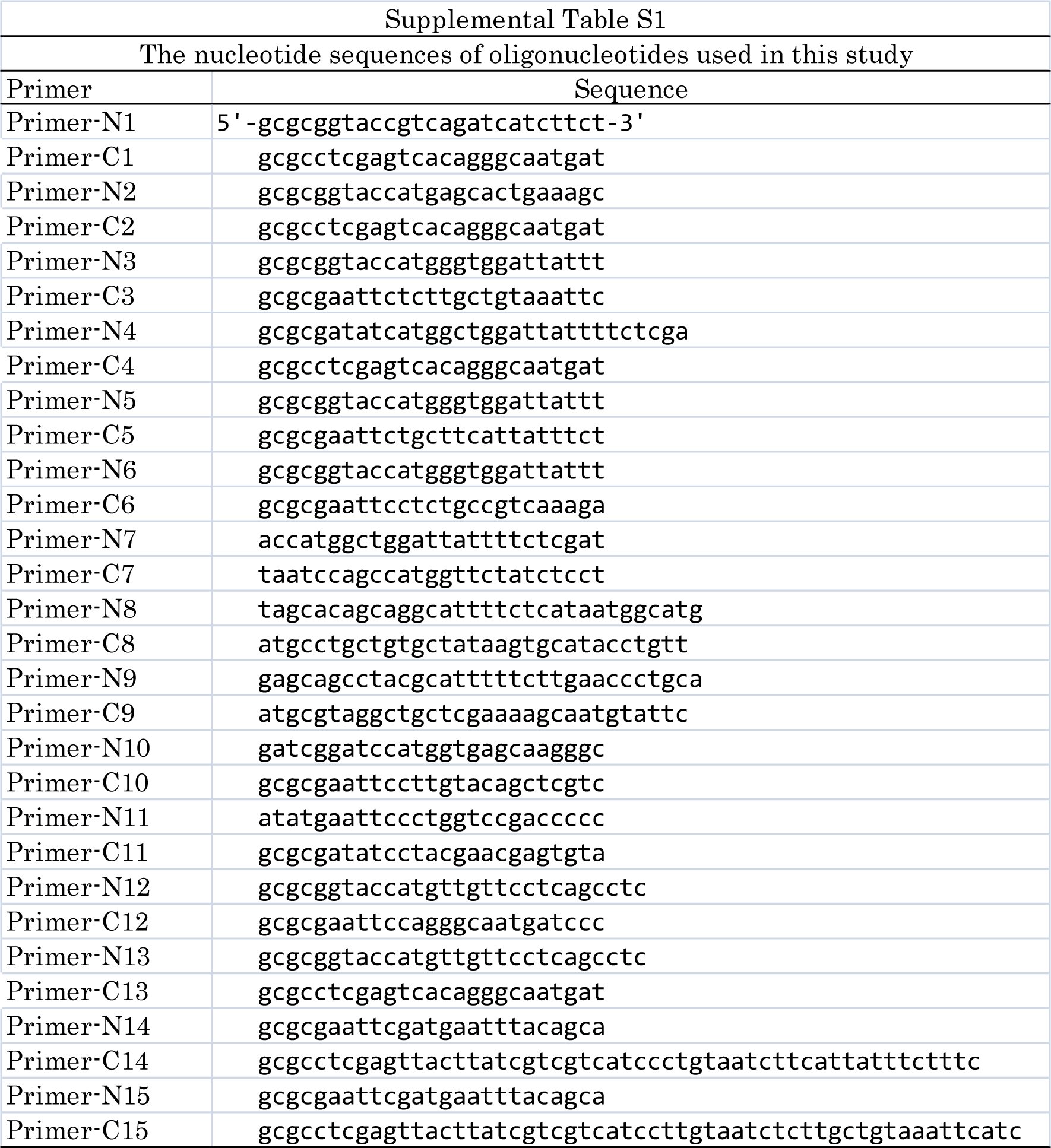

Supplement: Table S1 — The nucleotide sequences of oligonucleotides used in this study. (TIF) [file pone.0078235.s003.tif]

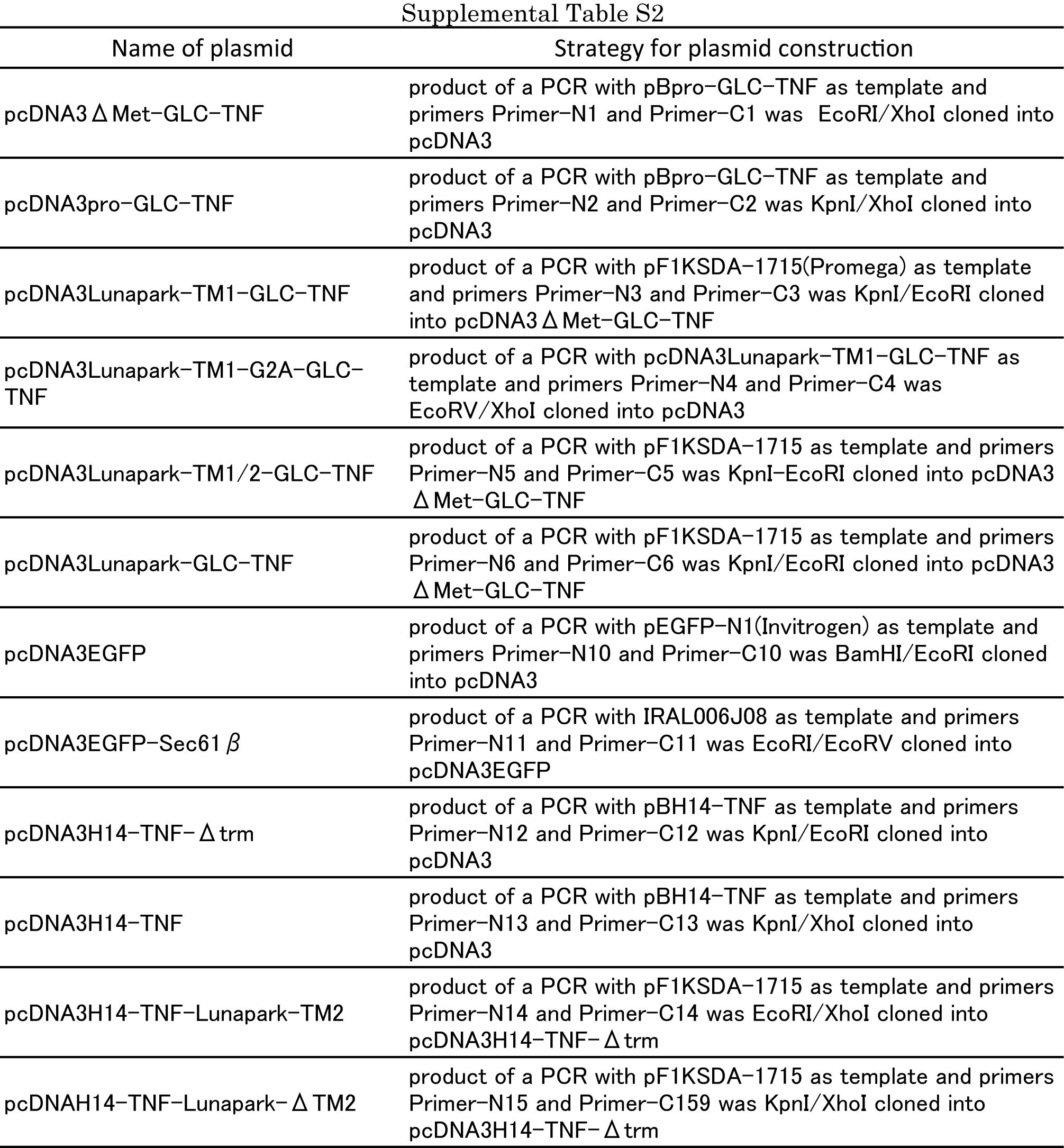

Supplement: Table S2 — The strategies for construction of plasmids used in this study. (TIF) [file pone.0078235.s004.tif]
